# Hospital practices and breastfeeding in Rio de Janeiro: data from the Nascer no Brasil II Survey

**DOI:** 10.11606/s1518-8787.2025059006603

**Published:** 2025-10-20

**Authors:** Ana Paula Esteves-Pereira, Katrini Guidolini Martinelli, Barbara Vasques da Silva Ayres, Marcia Leonardi Baldisserotto, Sonia Duarte de Azevedo Bittencourt, Claudio Heizer, Maria José Guardia Mattar, Paulo Blengini, Ricardo Leal Sabroza, Silvana Granado Nogueira da Gama, Maria do Carmo Leal

**Affiliations:** I Fundação Oswaldo Cruz. Escola Nacional de Saúde Pública. Departamento de Métodos Quantitativos em Saúde. Rio de Janeiro, RJ, Brasil; II Universidade Federal do Sul da Bahia. Centro de Formação em Saúde. Departamento de Medicina Social. Bahia, BA, Brasil; III Universidade Federal do Rio de Janeiro. Instituto de Psicologia. Departamento de Psicometria. Rio de Janeiro, RJ, Brasil; IV Universidade Cidade de São Paulo. Faculdade de Medicina. Internato de Neonatologia. São Paulo, SP, Brasil

**Keywords:** Breast Feeding, Social Determinants of Health, Parturition, Cohort Studies

## Abstract

**OBJECTIVE:**

To investigate the association between hospital practices and breastfeeding and exclusive breastfeeding at two months of age, and the main reasons for pre-lacteal feeding.

**METHODS:**

This is an analysis of the state of Rio de Janeiro, 2021–2023, from *Pesquisa Nascer no Brasil* II, a hospital-based cohort. We collected data during hospitalization for delivery and at two months of age. Logistic regression was used to analyse factors associated with breastfeeding and exclusive breastfeeding.

**RESULTS:**

959 mothers and babies were included and calibrated to represent 1,537 puerperal women at baseline. Around 60% of women breastfed at first hour of life, and almost 90% breastfed in the first 24 hours and practiced exclusive breastfeeding at hospital discharge. Around 95% of women were breastfeeding at two months, 61.4% exclusively. The chance of breastfeeding was significantly higher among women who had given birth in BFHI hospitals (OR = 2.35), had ≥ 12 years of schooling (OR = 1.96), had ≥ 3 previous births (OR = 4.35), intended to breastfeed for ≥ 1 year (OR = 1.58) and felt supported after discharge (OR = 6.91). While the chance of exclusive breastfeeding was higher among women with public funding for childbirth (OR = 1.33), with ≥ 16 years of schooling (OR = 2.27), who lived with a partner (OR = 1.33), and who intended to breastfeed for ≥ 1 year (OR = 1.49). Pre-lacteal feeding PLF at hospital discharge was negatively associated with breastfeeding (OR = 0.04) and exclusive breastfeeding (OR = 0.15), and was more frequent in the private sector (20.4%), among "early-term" (18.3%), and caesarean sections (15.6%).

**CONCLUSION:**

Breastfeeding promotion policies have been effective in almost universalizing breastfeeding at two months of age. However, in order to increase the prevalence of exclusive breastfeeding, it is necessary to expand and qualify support, management and information on exclusive breastfeeding, focus on vulnerable populations, reduce caesarean sections and improve hospital practices such as regulating the use of pre-lacteal feeding.

## INTRODUCTION

The World Health Organization and the Brazilian Ministry of Health recommend continued breastfeeding (BF) up to two years of age or beyond, and exclusive breastfeeding (EBF) until six months of age^1–3^. Globally, the prevalence of EBF up to six months increased from 38 to 48% between 2013 and 2023, approaching the World Health Assembly's target of 50% by 2025^4^. National surveys in Brazil indicated a rising trend in EBF prevalence among children under six months from 1986 to 2006 (2.9–37.1%), followed by stabilization in 2013 (36.6%)^5^ and renewed growth in 2019 (45.8%). The South and Southeast regions reported the highest prevalence rates in 2019, at 49.1 and 54.3%, respectively^6^.

In Brazil, the breastfeeding promotion policy comprises a comprehensive set of actions, including institutional practices such as the Baby-Friendly Hospital Initiative (*Iniciativa Hospital Amigo da Criança* – IHAC), professional training within the Brazilian Unified Health System (*Sistema Único de Saúde* – SUS), the creation and expansion of the human milk bank network, and the Breastfeed and Feed Brazil Strategy (*Estratégia Amamenta e Alimenta Brasil*), which promotes BF and healthy complementary feeding through primary health care^2^. Notable efforts also include intensive media-based educational campaigns and the active involvement of civil society, particularly organizations such as the International Network in Defense of the Right to Breastfeed, which monitor policy implementation and support breastfeeding promotion. The adoption of specific regulations and legislation, such as the Brazilian Standard for the Marketing of Food for Infants and Young Children, Nipples, Pacifiers, and Baby Bottles, along with maternity leave policies, complements this range of initiatives^7,8^.

The literature indicates that supportive workplace policies and practices for mothers (designated lactation spaces and scheduled breaks for milk expression) along with the implementation of the IHAC, skin-to-skin contact, kangaroo mother care, cup feeding in healthcare settings, and the continuity of care and support within community and family environments, are key actions that contribute to the large-scale protection, promotion, and support of breastfeeding^7,9,10^.

The negative impact of prelacteal feeding (PLF), which is the introduction of any liquids or foods other than breast milk during the first three days of life, on EBF up to six months of age is well established^11,12^. However, the influence of hospital practices on the continuation of breastfeeding remains underexplored and has yielded contradictory findings^12–14^.

Given this context, the present study aimed to investigate the association between hospital birth care practices and the continuation of BF and EBF at two months of age, as well as to identify the main reasons for the introduction of PLF. The central hypothesis is that certain hospital care practices, such as the provision of PLF without clinical indication, may adversely affect the continuation of BF and EBF.

## METHODS

This study is a nationally representative hospital-based cohort, with the first follow-up conducted at two months of age (*Nascer no Brasil II:* National Survey on Abortion, Childbirth, and Birth), conducted between 2021 and 2024.

### *Nascer no Brasil II* Survey

The population of the *Nascer no Brasil II* (NBII) Survey comprises women hospitalized for childbirth (live birth or stillbirth) or abortion in hospitals reporting 100 or more live births (LB) per year, according to the Live Birth Information System (*Sistema de Informações sobre Nascidos Vivos* – Sinasc). Exclusion criteria included triplet pregnancies, communication barriers (such as inability to understand Portuguese, hearing impairments, or severe mental illness), and hospitalizations for labor termination by court order.

A probabilistic sample was selected in two stages:

- Hospitals;
- Women and their newborns (NB).

Hospitals were stratified by macroregion, location (metropolitan or non-metropolitan), type (public, mixed, or private), and size (100–499 or ≥ 500 LB/year).

In the selected hospitals, women admitted for childbirth care were identified through available hospital records, including the hospital census and admissions book. Once identified, they were entered into a single list in chronological order by date of delivery and selected consecutively until the required sample size was reached — 30 postpartum women in facilities with 100–499 LB/year and 50 in those with ≥ 500 LB/year. Participants were approached during their hospital stay, while still in their hospital beds.

The hospital interview collected information on identification, education, income, obstetric history, anthropometric data, prenatal care, morbidities, medication use, intention to breastfeed, labor, and evaluation of the care received. Additionally, photographs were taken, and data were extracted from the prenatal care card and obstetric ultrasound examinations.

Data were extracted from hospital records of both women and NB, including information on prenatal care; hospital admission; care during labor, delivery, and birth; medications and interventions administered; maternal and neonatal morbidity; admission to the intensive care unit (ICU); and discharge conditions of women and NB. Data collection was conducted after hospital discharge or up to the 42^nd^ day of hospitalization for the woman, and after discharge or up to the 28^th^ day of NB hospitalization.

All postpartum women interviewed in the maternity ward who consented to subsequent contact were invited to participate in the two-month follow-up. This follow-up interview addressed topics including maternal morbidity after hospital discharge; use of outpatient health services; infant health; breastfeeding; satisfaction with care; post-traumatic stress disorder; mother-infant bonding; postpartum depression; and anxiety. Data collection was conducted either via telephone interview or through a self-administered questionnaire accessed via a link sent through WhatsApp, using the Oswaldo Cruz Foundation's Research Electronic Data Capture platform.

To assess the structure and care processes of obstetric and neonatology services, interviews were conducted with maternity hospital managers and coordinators in obstetrics, neonatology, epidemiology, and pharmacy. The protocols for the hospital phase and follow-up of the NBII study are detailed in Leal et al.^15^ and Miranda Theme-Filha et al.^16^.

### Study of the State of Rio de Janeiro

The sample of postpartum women in the state of Rio de Janeiro was calculated based on the proportion of cesarean sections in the state in 2019 (57%), with a 5% significance level and 90% power to detect a 7% difference. A design effect of 1.3 was applied, resulting in a planned minimum sample size of 1,350 postpartum women. To achieve this target, the sample size was increased from 50 to 90 postpartum women in public and mixed hospitals with ≥ 500 LB/year in the state. Post-hoc calculations indicated that this sample size provides sufficient power to detect absolute differences of 5% for outcomes with prevalences between 5 and 25%, corresponding to odds ratios (OR) greater than 1.3. In Rio de Janeiro, hospital interviews were conducted between November 2021 and June 2023 in 29 hospitals across 18 municipalities.

### Exclusion Criteria

For the present analysis, women hospitalized due to abortion and those presenting at least one risk criterion that could prevent or hinder breastfeeding were excluded. These criteria included fetal or neonatal death, twin birth, gestational age < 34 weeks, birth weight < 2,000 g, 5-minute Apgar score < 8, admission of the newborn to an inpatient unit/ICU, and maternal HIV-positive status. Additionally, infants older than 75 days at the time of follow-up were excluded. The newborns included in the final analysis are referred to as healthy newborns.

### Sample Weighting and Calibration

Basic sampling weights, baseline survey calibration (hospital interview), and calibration for follow-up losses (non-response) were applied. Basic sampling weights were calculated as the product of the inverse probabilities of inclusion at each sampling stage. Baseline survey calibration adjusted the distribution of LB according to hospital characteristics (location, type, and size) and maternal characteristics (age and type of delivery), based on data from Sinasc 2022. For calibration related to follow-up losses, the probability of response was modeled as a function of variables collected during the baseline survey, and these probabilities were used to generate non-response weight adjustments for participants who completed the follow-up.

### Outcome Variables

BF and EBF (no, yes) at two months of age were assessed using four 24-hour recall questions:

- From yesterday morning until this morning, did your baby breastfeed?;
- From…, did your baby have any other milk?;
- From…, did your baby have water, tea or juice?;
- From…, did your baby have any other food?

### Main Exposure Variables

Skin-to-skin contact in the delivery room, BF within the first hour of life, and BF within the first 24 hours were reported by postpartum women during the hospital interview. PLF at hospital discharge and post-discharge BF support were reported during the first follow-up. PLF at hospital discharge was defined as cases in which the newborn was not exclusively breastfed at the time of discharge from the maternity ward. All variables were dichotomized (no, yes).

### Other Exposure Variables/Covariates

Hospital characteristics included the classification of facilities according to their status within IHAC, grouped into three categories:

- Hospitals with fully implemented IHAC;
- Hospitals in the process of accreditation;
- Hospitals that did not fit into any of the categories.

In addition, the presence of exclusive rooming-in was considered (no, yes). This was defined as hospitals having exclusively pre-delivery, delivery, and post-delivery rooms, or obstetric beds with rooming-in. Both pieces of information were obtained through interviews with the facility managers.

Sociodemographic characteristics included: source of payment for childbirth (public or private); self-reported skin color (white, black, brown, Asian, or Indigenous); maternal age (< 20, 20–34, or ≥ 35 years); years of education (≤ 8, 9–11, 12–15, ≥ 16, or ≥ 12 years); cohabitation with a partner (no, yes); paid employment (no, yes); and parity (nulliparous, one or two births, or ≥ three births) or (0–2 births or ≥ three births). Different categorizations for years of education and parity were used as appropriate for each outcome.

Women who gave birth in public or private hospitals affiliated with SUS, without hospitalization costs covered by a health plan, were classified as having a public source of payment. Those who gave birth in private hospitals, with costs covered either by a health plan or through direct out-of-pocket payment, were classified as having a private source of payment.

Prenatal and delivery data included whether the infant's caregiver received information during prenatal care regarding the importance of breastfeeding within the first hour of life and the practice of EBF until six months of age (no: zero or only one of these topics was addressed, yes: both were covered); intention to breastfeed (< 6 months, 6 months to < 1 year, 1 to < 2 years, ≥ 2 years); gestational age at birth in completed weeks (34–36, 37–38, ≥ 39); and type of delivery (vaginal, including forceps and vacuum extraction; cesarean section). Sociodemographic and prenatal characteristics were reported by postpartum women during the hospital interview. Gestational age was primarily determined based on early ultrasound (performed between 6 and 13 weeks of gestation), as documented in hospital records and the pregnant women's health handbooks. Type of delivery and variables used to define risk criteria were extracted from hospital records.

### Data Analysis

Descriptive analyses were stratified by the source of payment for delivery and the type of delivery. Proportions and p-values were estimated using Pearson's χ² test. Logistic regression was employed to analyze factors associated with BF and EBF. A theoretical model was applied to classify variables according to their hierarchical level:

- Distal (level 1): hospital characteristics, sociodemographics factors, and parity;
- Intermediate 1 (level 2): prenatal and delivery characteristics;
- Intermediate 2 (level 3): hospital practices;
- Proximal (level 4): post-hospital discharge support for BF.

At each level, variables were selected for inclusion in the adjusted model based on a significance threshold of p < 0.20, with the exception of source of payment for delivery, type of delivery, and gestational age at birth. These variables were included in the model regardless of their p-values due to their critical relevance^17^. Variables that showed a statistically significant association with the outcomes (p < 0.05) were retained in the final regression model after adjustment for variables at the same and higher hierarchical levels. Crude and adjusted odds ratios were calculated, along with their respective 95% confidence intervals (95%CI). Additionally, analysis of the reasons for PLF was stratified by source of payment for delivery, type of delivery, and gestational age at birth, with proportions and corresponding 95%CIs estimated. All analyses accounted for the sampling design, including weighting and calibration, and were conducted using IBM Statistical Package for the Social Sciences Statistics for Windows, Version 23.0 (IBM Corp., Armonk, NY, USA).

## RESULTS

A total of 1,762 postpartum women were interviewed in maternity hospitals in the state of Rio de Janeiro, of whom 225 (13%) were excluded based on the predefined risk criteria. Among the remaining 1,537 women, there were 372 losses to follow-up and 167 refusals, resulting in a follow-up rate of 65%. An additional 39 infants older than 75 days at the time of follow-up were excluded. The final analysis included 959 mothers and infants aged 45 to 75 days, with calibration applied to represent the original sample of 1,537 postpartum women. The mean age of the infants was 61 days, with the 25^th^ and 75^th^ percentiles at 56 and 65 days, respectively. No differences were observed in exposure or outcome variables across age distribution (data not shown).

Significant differences were observed in hospital and sociodemographic characteristics based on source of delivery financing and type of delivery. While 51% of women in the public sector gave birth in hospitals with an implemented IHAC, this proportion was only 17% among those in the private sector. Exclusive rooming-in was highly prevalent across both financing groups, occurring in over 85% of cases. Women with privately funded deliveries and those who underwent cesarean sections were more likely to be white, older, have higher educational attainment, be employed, live with a partner, and have fewer children (Table 1).

**Table 1:** Hospital and sociodemographic characteristics of women according to delivery funding source and type of delivery. *Nascer no Brasil* II Survey in the state of Rio de Janeiro, 2021–2023.

Total number of women			Delivery funding source			Type of delivery			Total 1,537
			Public 1,131	Private 406	p-value^a^	Vaginal 634	Cesarean 903	p-value^a^	
Age at follow-up (days)									
	45 to 60		48.2	49.1	0.848	50.4	47.2	0.399	48.6
	61 to 75		51.8	50.9		49.6	52.8		51.4
Hospital characteristics									
	*Iniciativa Hospital Amigo da Criança*								
		No	10.6	15.9		9.4	13.9		12.0
		In process^b^	38.4	67.1	0.04	35.0	53.6	0.03	46.0
		Yes	51.0	17.0		55.6	32.5		42.0
	Exclusive rooming-in								
		No	9.7	12.5	0.77	5.3	14.1	0.32	10.5
		Yes	90.3	87.5		94.7	85.9		89.5
Sociodemographic characteristics									
	Skin color								
		White	23.4	51.6		27.6	33.1		30.8
		Black	25.5	9.4	< 0.001	23.5	19.6	< 0.001	21.2
		Brown	51.2	39.0		48.9	47.3		48.0
	Mother's age (years)								
		10 to 19	13.6	1.7		15.4	6.9		10.4
		20 to 34	73.7	68.0	< 0.001	72.3	72.1	< 0.001	72.2
		35 or more	12.7	30.4		12.2	21.0		17.4
	Education level (years)								
		≤ 8 (incomplete primary education)	18.8	1.0		18.7	10.9		14.1
		9 to 11 (complete primary education)	35.7	8.4	< 0.001	31.9	26.1	< 0.001	28.5
		12 to 15 (complete secondary education)	40.3	43.1		42.1	40.3		41.1
		≥ 16 (complete higher education)	5.1	47.5		7.3	22.6		16.3
	Lives with a partner								
		No	22.9	5.7	< 0.001	22.7	15.3	< 0.001	18.3
		Yes	77.1	94.3		77.3	84.7		81.7
	Employed								
		No	62.6	32.6	< 0.001	63.2	48.7	< 0.001	54.7
		Yes	37.4	67.4		36.8	51.3		45.3
	Parity								
		Primiparous	39.5	57.1		40.9	46.4		44.2
		One or two previous births	48.5	39.4	< 0.001	44.3	47.4	< 0.001	46.1
		Three or more previous births	12.0	3.5		14.8	6.2		9.7

a Test χ^2^.

b Hospitals undergoing accreditation for *Iniciativa Hospital Amigo da Criança* or with updated protocols for newborn care in the delivery room, including early skin-to-skin contact and breastfeeding.

Prenatal guidance on breastfeeding was reported by just over half of the women (52.9%), with higher prevalence in the private sector (64.1%). More than 40% intended to breastfeed beyond two years, and 60% planned to do so for at least one year. Skin-to-skin contact in the delivery room and BF within the first hour of life were reported in approximately 60% of cases. However, BF within the first 24 hours and EBF at hospital discharge reached nearly 90%. These hospital practices were more frequent in the public sector and among women who had vaginal deliveries. Approximately 85% of women reported receiving breastfeeding support, and 95.2% were breastfeeding at two months postpartum, with 61.4% practicing EBF. No significant differences were observed in these outcomes by type of delivery or source of financing (Table 2).

**Table 2:** Guidance, intention, hospital practices, post-discharge support, and breastfeeding at two months of age according to delivery funding and type of birth. *Nascer no Brasil* II survey, Rio de Janeiro State, 2021–2023.

			Delivery funding source			Type of delivery			Total
			Public	Private	p-value^a^	Vaginal	Cesarean	p-value^a^	
Total number of women			1,131	406		634	903		1,537
Received breastfeeding guidance during prenatal care									
	No		51.1	35.9	0.047	51.1	44.3	0.124	47.1
	Yes		48.9	64.1		48.9	55.7		52.9
Intended to breastfeed									
	Yes		96.6	98.0	0.190	96.2	97.4	0.162	96.7
		< 6 months	4.7	3.3		3.7	4.8		4.3
		6 months to < 1 year	26.1	27.3		28.6	24.8		26.4
		1 to < 2 years	19.0	16.6	0.581	18.4	18.3	0.524	18.3
		≥ 2 years	41.9	42.2		39.9	43.4		42.0
	Did not think about the duration		8.3	10.6		9.4	8.7		9.0
Hospital practices									
	Skin-to-skin contact in the delivery room		57.8	66.1	0.232	80.7	45.4	< 0.001	60.0
	Breastfeeding within the first hour of life		65.6	51.7	0.041	78.3	50.2	< 0.001	61.9
	Breastfeeding within the first 24 hours of life		88.7	88.2	0.887	92.9	85.4	< 0.001	88.5
	Prelacteal feeding at hospital discharge		11.0	20.4	0.028	10.3	15.6	0.044	13.4
Post-discharge breastfeeding support									
	No		16.3	12.9	0.374	14.8	15.9	0.623	15.4
	Yes		83.7	87.1		85.2	84.1		84.6
Breastfeeding at 2 months of age^b^									
	Yes		94.8	96.5		96.0	94.7		95.2
		Exclusive	60.9	64.2		64.0	60.2		61.4
		Non-exclusive	33.9	32.3	0.303	32.0	34.5	0.683	33.8
	No		5.2	3.5		4.0	5.2		4.8

a Test χ^2^.

b Breastfeeding between 45 and 75 days of life, mean and median age of 61 days.

The odds of BF were higher among women who delivered in IHAC-accredited hospitals (OR = 2.35; 95%CI 1.04–5.32), had ≥ 12 years of education (OR = 1.96; 95%CI 1.08–3.58), had three or more previous births (OR = 4.35; 95%CI 1.06–17.83), and intended to breastfeed for ≥ one year (OR = 1.58; 95%CI 1.09–2.31). The provision of PLF at hospital discharge was negatively associated with BF (OR = 0.04; 95%CI 0.02–0.13). Women who reported receiving breastfeeding support after discharge had significantly higher odds of BF (OR = 6.91; 95%CI 3.57–13.35) (Table 3).

**Table 3:** Factors associated with breastfeeding at two months of age. *Nascer no Brasil II* Survey in the state of Rio de Janeiro, 2021–2023.

			Breastfeeding^a^		p-value^b^	Adjusted regression^c^	
			No (%)	Yes (%)		OR	95%CI
Total women (n = 1,537)			4.8	95.2	-	-	-
	Age at follow-up (days)						
		45 to 60	4.9	95.1		ref	-
		61 to 75	5.0	95.0	0.922	0.98	0.62–1.54
Level 1							
	Birth financing						
		Public	5.0	95.0		1.64	0.56–4.76
		Private	4.4	95.6	0.647	ref	-
	*Iniciativa Hospital Amigo da Criança*						
		No	7.9	92.1		ref	-
		In process^d^	4.6	95.4	0.196	2.04	0.86–4.81
		Yes	4.2	95.8		2.35	1.04–5.32
	Exclusive rooming-in						
		No	6.8	93.2		ref	-
		Yes	4.6	95.4	0.119	1.73	0.94–3.30
	Skin color						
		White	5.0	95.0		ref	-
		Black	5.9	94.1	0.662	0.87	0.18–4.11
		Brown	4.1	95.9		1.32	0.71–2.44
	Mother's age (years)						
		10 to 19	4.5	95.5		1.61	0.13–0.63
		20 to 34	5.4	94.6	0.483	ref	-
		35 or more	2.9	97.1		1.78	0.56–5.70
	Education (years)						
		≤ 8	6.6	93.4		ref	-
		9 to 11	4.6	95.4	0.550	1.66	0.36–7.64
		≥ 12 years	4.4	95.6		1.96	1.08– 3.58
	Lives with partner						
		No	5.2	94.8		ref	-
		Yes	4.8	95.2	0.870	1.08	0.31–3.77
	Employment						
		No	5.3	94.7		ref	-
		Yes	4.3	95.7	0.465	1.39	0.72–2.69
	Parity						
		0 to two previous births	5.2	94.8		ref	-
		Three or more previous births	1.7	98.3	0.200	4.35	1.06–7.83
Level 2							
	Received breastfeeding guidance during prenatal care						
		No	5.3	94.7	0.680	ref	-
		Yes	4.5	95.5	1.09	0.53–2.26
	Intention to breastfeed for one year or more						
		No	6.3	93.7		ref	-
		Yes	3.9	96.1	0.004	1.58	1.09–2.31
	Type of delivery						
		Vaginal	4.1	95.9		1.37	0.55–3.43
		Cesarean	5.4	94.6	0.463	ref	-
	Gestational age (weeks)						
		34–36	1.4	98.6		4.06	0.45–37.07
		37–38	3.9	96.1	0.157	1.46	0.83–2.56
		≥ 39	5.7	94.3		ref	-
Hospital practices (level 3)							
	Skin-to-skin contact immediately after birth						
		No	6.1	93.9		ref	-
		Yes	3.9	96.1	0.313	1.60	0.61–4.19
	Breastfeeding within the first hour of life						
		No	5.5	94.5		ref	-
		Yes	4.4	95.6	0.595	0.75	0.25–2.19
	Breastfeeding within the first 24 hours of life						
		No	7.1	92.9		ref	-
		Yes	4.5	95.5	0.377	1.02	0.30–3.48
	Pre-lacteal feeding at hospital discharge						
		No	2.3	97.7		ref	-
		Yes	21.1	78.9	< 0.001	0.04	0.02– 0.13
Level 4							
	Post-discharge breastfeeding support						
		No	14.5	85.5		ref	-
		Yes	2.8	97.2	< 0.001	6.91	3.57–13.35

95%CI: 95% confidence interval; OR: odds ratio; PLF: prelacteal feeding; ref: reference.

a Breastfeeding between 45 and 75 days of life, mean and median of 61 days of life.

b Test ○S^2^.

c Level 1 adjusted for childbirth funding, *Iniciativa Hospital Amigo da Criança*, education, and parity. Levels 2 and 3 adjusted for childbirth funding, *Iniciativa Hospital Amigo da Criança*, education, parity, intention to breastfeed for one year or more, type of delivery, and gestational age. Level 4 adjusted for childbirth funding, *Iniciativa Hospital Amigo da Criança*, education, parity, intention to breastfeed for one year or more, type of delivery, gestational age, and PLF at hospital discharge.

d Hospitals in the process of accreditation for *Iniciativa Hospital Amigo da Criança* or with updated protocols for newborn care in the delivery room, including early skin-to-skin contact and breastfeeding.

The odds of EBF were higher among women with publicly funded childbirth (OR = 1.33; 95%CI 1.02–1.72), those with ≥ 16 years of education (OR = 2.27; 95%CI 1.10–4.71), those living with a partner (OR = 1.33; 95%CI 1.06–1.67), and those intending to breastfeed for ≥ one year (OR = 1.49; 95%CI 1.05–2.11). The provision of PLF at hospital discharge was negatively associated with EBF (OR = 0.15; 95%CI 0.09–0.28) (Table 4).

**Table 4:** Factors associated with exclusive breastfeeding at two months of age. *Nascer no Brasil* II Survey in the state of Rio de Janeiro, 2021–2023.

			Exclusive breastfeeding^a^		p-value^b^	Adjusted regression^c^	
			No (%)	Yes (%)		OR	95%CI
Total women (n = 1,537)			38.6	61.4	-	-	-
	Age at follow-up (days)						
		45 to 60	41.5	58.5		ref	-
		61 to 75	35.7	64.3	0.248	1.27	0.83–1.96
Level 1							
	Funding						
		Public	38.9	61.1		1.33	1.02–1.72
		Private	37.5	62.5	0.653	ref	-
	*Iniciativa Hospital Amigo da Criança*						
		No	36.3	63.7		ref	-
		In process^d^	38.7	61.3	0.819	0.87	0.56–1.33
		Yes	39.1	60.9		0.91	0.57–1.44
	Exclusive rooming-in						
		No	40.0	60.0		ref	-
		Yes	38.4	61.6	0.799	0.97	0.57–1.65
	Skin color						
		White	41.2	58.8		ref	-
		Black	38.3	61.7	0.540	1.15	0.81–1.63
		Brown	37.0	63.0		1.22	0.82–1.82
	Mother's age (years)						
		10 to 19	48.0	52.0		0.76	0.47–1.24
		20 to 34	37.9	62.1	0.242	ref	-
		35 or more	35.8	64.2		1.10	0.65–1.85
	Education (years)						
		≤ 8	46.9	53.1		ref	-
		9 to 11	43.0	57.0		1.11	0.61–2.03
		12 to 15	35.3	64.7	0.047	1.61	0.84–3.09
		≥ 16	31.5	68.5		2.27	1.10–4.71
	Lives with partner						
		No	46.5	53.5		ref	-
		Yes	36.9	63.1	0.001	1.33	1.06–1.67
	Employment						
		No	39.1	60.9		ref	-
		Yes	37.9	62.1	0.764	0.91	0.68–1.21
	Parity						
		Primiparous	39.4	60.6		1.20	0.74–1.94
		One or two previous births	35.2	64.8	0.074	1.57	0.86–2.85
		Three or more previous births	50.8	49.2		ref	-
Level 2							
	Received breastfeeding counseling during prenatal care						
		No	39.3	60.7	0.768	ref	-
		Yes	37.9	62.1	1.04	0.79–1.37
	Intention to breastfeed for one year or more						
		No	45.0	55.0		ref	-
		Yes	34.5	65.5	0.028	1.49	1.05–2.11
	Type of delivery						
		Vaginal	35.4	64.6		1.43	0.83–2.47
		Cesarean	40.7	59.3	0.401	ref	-
	Gestational age (weeks)						
		34–36	38.2	61.8		0.95	0.53–1.69
		37–38	39.3	60.7	0.942	0.93	0.70–1.24
		≥ 39	38.2	61.8		ref	-
Hospital practices (level 3)							
	Skin-to-skin contact immediately after birth						
		No	42.5	57.5		ref	-
		Yes	35.6	64.4	0.335	1.23	0.70–2.17
	Breastfeeding within the first hour of life						
		No	41.2	58.8		ref	-
		Yes	36.7	63.3	0.275	1.07	0.78–1.47
	Breastfeeding within the first 24 hours of life						
		No	46.1	53.9		ref	-
		Yes	37.5	62.5	0.151	1.23	0.75–2.01
	Pre-lacteal feeding at hospital discharge						
		No	32.9	67.1		ref	-
		Yes	75.1	24.9	< 0.001	0.15	0.09–0.28
Level 4							
	Post-discharge breastfeeding support						
		No	42.2	57.8		ref	-
		Yes	37.7	62.3	0.450	1.20	0.78–1.86

95%CI: 95% confidence interval; OR: odds ratio; PLF: prelacteal feeding; ref: reference.

a Exclusive breastfeeding between 45 and 75 days of life, with a mean and median of 61 days of life.

b Test χ^2^.

c Level 1 adjusted for birth funding, education, living with a partner, and parity. Levels 2 and 3 adjusted for birth funding, education, living with a partner, parity, intention to breastfeed for one year or more, type of delivery, and gestational age. Level 4 adjusted for birth funding, education, living with a partner, parity, intention to breastfeed for one year or more, type of delivery, gestational age, and pre-lacteal feeding at hospital discharge.

d Hospitals in the process of accreditation for *Iniciativa Hospital Amigo da Criança* or with updated protocols for newborn care in the delivery room, including early skin-to-skin contact and breastfeeding.

The provision of PLF at hospital discharge was more frequent in the private sector (20.4%), among early-term births (37–38 completed weeks; 18.3%), and following cesarean sections (15.6%). The primary reason reported by postpartum women was insufficient milk and/or difficulties with latching, accounting for over 70% of cases (Table 5).

**Table 5:** Prevalence and reasons for pre-lacteal feeding at hospital discharge according to birth funding, type of delivery, and gestational age at birth, *Nascer no Brasil* II Survey in the state of Rio de Janeiro, 2021–2023.

Total women		Funding		Type of delivery		Gestational age in complete weeks			Total
		Public	Private	Vaginal	Cesarean	34–36	37–38	≥ 39	
		1,131	406	634	903	98	503	936	1,537
		%	%	%	%	%	%	%	%
		(95%CI)	(95%CI)	(95%CI)	(95%CI)	(95%CI)	(95%CI)	(95%CI)	(95%CI)
Prelacteal feeding at hospital discharge		11.0 (8.6–13.9)	20.4 (14.4–28.1)	10.3 (7.3–14.3)	15.6 (12.5–19.2)	10.5 (3.7–26.3)	18.3 (14.3–23.0)	11.1 (9.0–13.6)	13.4 (11.1–16.1)
Reasons									
	Newborn had a health problem	0.8 (0.5–1.6)	2.1 (1.0–4.5)	1.1 (0.4–2.7)	1.2 (0.6–2.4)	1.9 (0.5–7.6)	1.3 (0.6–2.7)	1.0 (0.5–2.1)	1.1 (0.7–1.9)
	Mother had a health problem	0.6 (0.3–1.5)	1.7 (0.5–5.3)	0.3 (0.1–1.2)	1.3 (0.6–2.9)	0.0 -	0.7 (0.2–1.8)	1.1 (0.4–2.7)	0.9 (0.4–1.9)
	Insufficient milk and/or latching difficulties	7.8 (5.2–11.6)	14.5 (10.2–20.2)	7.3 (4.7–11.1)	11.1 (8.4–14.3)	7.0 (2.8–16.3)	12.6 (9.1–17.3)	8.1 (6.0–10.9)	9.5 (7.4–12.1)
	Hospital routine	1.0 (0.3–2.9)	1.7 (0.6–4.6)	1.0 (0.3–3.3)	1.2 (0.5–2.8)	0.0 -	2.4 (0.9–6.2)	0.6 (0.2–2.1)	1.1 (0.5–2.5)
	Other reason	2.6 (1.7–3.9)	4.5 (2.4–8.2)	2.9 (1.7–4.9)	3.2 (1.9–5.2)	2.2 (0.6–7.5)	5.9 (3.8–9.3)	1.6 (0.8–3.5)	3.0 (2.2–4.2)

95%CI: 95% confidence interval.

## DISCUSSION

Hospital characteristics, sociodemographic factors, and variables related to BF intention and support were strongly associated with the continuation of BF. Skin-to-skin contact in the delivery room and breastfeeding within the first hour of life occurred less frequently than expected. In contrast, breastfeeding within the first 24 hours was nearly universal, with minimal differences by type of delivery. However, none of these three practices were associated with continued BF. Conversely, the provision of PLF at hospital discharge significantly reduced the likelihood of both BF and EBF at two months of age.

In low- and middle-income countries^11^ and among Amazonian children from Cruzeiro do Sul (Acre)^12^, PLF has been associated with shorter durations of EBF and increased formula consumption in children under six months of age. In addition to this negative impact, our findings indicate that insufficient milk production and/or latching difficulties were the main reasons reported by mothers of healthy newborns in the state of Rio de Janeiro. Globally, approximately half of mothers cite perceived insufficient milk as the primary reason for introducing infant foods during the first months of life and for early cessation of BF, although this issue can typically be prevented or effectively managed with appropriate support^9^.

Not only was PLF more frequent in the private sector, early term births, and cesarean sections, but so were the reasons for its use, namely, insufficient milk production and/or difficulties with latching. Cesarean sections have previously been associated with increased PLF in Latin American and Caribbean countries^18^, as well as with lower rates of several BF indicators, including early initiation^19,20^, BF at hospital discharge^20^, and EBF before six months of age^20^. The main factors contributing to delayed BF after cesarean delivery include postoperative pain^21,22^, limited mobility^22^, and delayed onset of lactogenesis^21,23,24^. Brazil presents extremely high rates of cesarean deliveries (58%, according to Sinasc 2022) and early term births (approximately 35%), both of which are associated with reduced chances of BF in the first hour of life and of EBF during hospitalization^17^. In this context, prioritizing early BF initiation, combined with physical and emotional support from health professionals and family members, is essential to stimulate oxytocin and prolactin release, enhance maternal confidence, and strengthen the mother-infant bond.

The importance of IHAC in promoting breastfeeding is well established^20^. In the state of Rio de Janeiro, women who gave birth in IHAC-accredited hospitals were more likely to continue BF, although some individual practices were not independently associated with BF outcomes. This finding suggests that other components of the IHAC, such as restricted PLF, the availability of support services during hospitalization, and referrals to post-discharge support groups, play a crucial role in sustaining BF. A prospective study conducted in Shanghai found that mothers who received high-quality support during hospitalization were more likely to maintain EBF at both hospital discharge and at six months^25^. Similarly, a study involving postpartum women in the United States demonstrated that hospitals adhering to at least six of the IHAC's Ten Steps significantly increased the likelihood of EBF, underscoring the institutional importance of these practices^26^.

Mothers whose deliveries were publicly funded were more likely to maintain EBF. In the public sector, the higher prevalence of IHAC accreditation is associated with reduced influence from the infant formula industry, thereby decreasing the prescription of breast milk substitutes. Furthermore, access to ongoing support from multidisciplinary healthcare teams and community-based initiatives, such as the Family Health Strategy and the Breastfeeding and Feeding Brazil Strategy, may have contributed to this outcome.

Maternal education and the intention to breastfeed for at least one year were identified as highly influential factors, likely due to improved access to information about breastfeeding practices and benefits, greater access to healthcare services, increased autonomy in decision-making, and stronger family and social support. These findings underscore the importance of prenatal attitudes and intentions in shaping breastfeeding behavior. A study on knowledge, practices, and attitudes found that breastfeeding knowledge, attitude, subjective norms, and perceived behavioral control were positively associated with EBF at four months. Common barriers included low confidence in breastfeeding, lactation difficulties, and the misperception of insufficient milk supply^27^. A systematic review further concluded that unstable infant behaviors, particularly persistent crying, often lead parents to believe that formula supplementation is necessary^28^.

Women with high parity were more likely to maintain BF, suggesting that prior experience with motherhood and breastfeeding may contribute to greater success in current BF, possibly due to increased knowledge, confidence, and the use of more effective coping strategies^29^. Although EBF was less prevalent among these women, the difference was not statistically significant. A possible explanation is that managing EBF with a larger number of children may present additional challenges, requiring more time, organization, and support.

Living with a partner was associated with a higher probability of EBF, but not with BF in general. This finding suggests that support within the immediate family may play a crucial role in maintaining EBF by helping to prevent the introduction of other liquids or foods in addition to breast milk. Because EBF requires strict adherence to feeding routines, it may demand greater commitment and coordination among family members. In contrast, women who reported feeling supported had a higher prevalence of BF, though this was not observed for EBF. One possible explanation is that perceived support may stem from networks outside the family, such as social networks or support from healthcare professionals and services, which can be effective in preventing early weaning but may exert less influence on the maintenance of EBF, which requires more sustained, daily adaptation.

Among the strengths of the study are its design and sample size, which ensure representativeness of the state of Rio de Janeiro, as well as the standardization of data collection and the low refusal rate during hospital interviews. These factors contributed to the robustness of the findings. A limitation of the study is the simultaneous collection of exposure variables to PLF at hospital discharge and breastfeeding support after discharge, and outcome measurements, which may have introduced reverse causality bias.

In conclusion, the comprehensive breastfeeding promotion policy implemented in Brazil has been effective in achieving near-universal breastfeeding at 2 months of age among healthy newborns in the state of Rio de Janeiro. However, increasing the prevalence of exclusive breastfeeding requires progress in several key areas. Priorities identified in this study include: delivering evidence-based, industry-independent information to women and their families during prenatal, childbirth, and postpartum care, with particular focus on vulnerable groups (low education, primiparous mothers, and those without a partner); implementing strategies to reduce cesarean sections, particularly before 39 weeks of gestation; training health managers and professionals to provide effective support for breastfeeding difficulties both during hospitalization and postpartum; and enhancing adherence to hospital breastfeeding promotion practices, including stricter regulation of PLF, especially in the private sector.

For future research, it is recommended to deepen the investigation of factors influencing PLF, considering not only hospital practices but also maternal perceptions of milk insufficiency and difficulties with latching. Additionally, studies should explore effective strategies to reduce barriers to breastfeeding in specific contexts, such as cesarean sections, early-term and preterm births, and assess the role of support networks, both familial and community-based, in the continuation of EBF. Finally, it is essential to investigate the effectiveness of public breastfeeding support programs, considering their implementation in the private sector, as well as the impact of regulatory policies on the prescription and distribution of infant formulas.

## Data Availability

The datasets generated and/or analyzed during the study are available from the corresponding author upon request.
