# Supplementary material for: Hospital practices and breastfeeding in Rio de Janeiro: data from the Nascer no Brasil II Survey
Source: Rev Saude Publica. 2025 Oct 20;59(Suppl 1):e7s. doi: 10.11606/s1518-8787.2025059006603 (PMC12539639; doi:10.11606/s1518-8787.2025059006603)
Supplement: Translation

# Translation: Práticas hospitalares e amamentação no Rio de Janeiro: dados da Pesquisa Nascer no Brasil II

**Authors:** Ana Paula Esteves-Pereira, Katrini Guidolini Martinelli, Barbara Vasques da Silva Ayres, Marcia Leonardi Baldisserotto, Sonia Duarte de Azevedo Bittencourt, Claudio Heizer, Maria José Guardia Mattar, Paulo Blengini, Ricardo Leal Sabroza, Silvana Granado Nogueira da Gama, Maria do Carmo Leal

**Affiliations:** I Fundação Oswaldo Cruz. Escola Nacional de Saúde Pública. Departamento de Métodos Quantitativos em Saúde. Rio de Janeiro, RJ, Brasil; II Universidade Federal do Sul da Bahia. Centro de Formação em Saúde. Departamento de Medicina Social. Bahia, BA, Brasil; III Universidade Federal do Rio de Janeiro. Instituto de Psicologia. Departamento de Psicometria. Rio de Janeiro, RJ, Brasil; IV Universidade Cidade de São Paulo. Faculdade de Medicina. Internato de Neonatologia. São Paulo, SP, Brasil

**Keywords:** Aleitamento Materno, Determinantes Sociais da Saúde, Parto, Estudos de Coortes

## Abstract

**OBJETIVO:**

Investigar a associação entre as práticas hospitalares e o aleitamento materno e o aleitamento materno exclusivo aos 2 meses de idade e os principais motivos para a alimentação pré-láctea.

**MÉTODOS:**

Coorte de base hospitalar, um recorte da Pesquisa Nascer no Brasil II no estado do Rio de Janeiro, 2021–2023. Coletamos dados durante a internação para o parto e aos 2 meses de idade. Para a análise dos fatores associados ao aleitamento materno e ao aleitamento materno exclusivo, utilizamos regressão logística.

**RESULTADOS:**

Foram incluídos 959 mães e bebês, calibrados para representar 1.537 puérperas do *baseline*. Cerca de 60% das mulheres amamentaram na primeira hora de vida, e quase 90% amamentaram nas primeiras 24 horas e praticavam aleitamento materno exclusivo na alta hospitalar. Cerca de 95% das mulheres amamentavam aos 2 meses, sendo 61,4% exclusivamente. A chance de aleitamento materno foi significativamente maior entre mulheres com parto em hospitais Iniciativa Hospital Amigo da Criança (*odds ratio* — OR = 2,35), com ≥ 12 anos de estudo (OR = 1,96), com ≥ três partos anteriores (OR = 4,35), que tinham a intenção de amamentar ≥ um ano (OR = 1,58) e se sentiram apoiadas após a alta (OR = 6,91), enquanto a chance de aleitamento materno exclusivo foi maior nas mulheres com financiamento público do parto (OR = 1,33), com ≥ 16 anos de estudo (OR = 2,27), que viviam com companheiro(a) (OR = 1,33) e que tinham a intenção de amamentar ≥ 1 ano (OR = 1,49). A alimentação pré-láctea na alta hospitalar se mostrou negativamente associada ao aleitamento materno (OR = 0,04) e ao aleitamento materno exclusivo (OR = 0,15), sendo mais frequente no setor privado (20,4%), entre termo precoce (18,3%), e nas cesarianas (15,6%).

**CONCLUSÃO:**

A política de promoção do aleitamento materno tem sido eficaz na quase universalização do aleitamento materno aos 2 meses de idade, entretanto para aumentar a prevalência do aleitamento materno exclusivo é necessário ampliar e qualificar o apoio, o manejo e as informações sobre o aleitamento materno exclusivo, focar nas populações vulneráveis, reduzir cesarianas e aprimorar práticas hospitalares como a regulação do uso de alimentação pré-láctea.

## INTRODUÇÃO

É recomendação da Organização Mundial da Saúde e do Ministério da Saúde o aleitamento materno (AM) continuado até os 2 anos ou mais, e o aleitamento materno exclusivo (AME) até os 6 meses de vida^1–3^. O percentual de AME mundial até os 6 meses aumentou de 38 para 48% entre 2013 e 2023, valor próximo à meta de 50% da Assembleia Mundial da Saúde para 2025^4^. Inquéritos nacionais brasileiros mostraram que a prevalência de AME entre os menores de 6 meses apresentou tendência crescente para o período de 1986 a 2006 (2,9–37,1%), de estabilização em 2013 (36,6%)^5^ e retomada do crescimento em 2019 (45,8%), com as regiões Sul e Sudeste apresentando os maiores percentuais, 49,1 e 54,3%, respectivamente^6^.

No Brasil, a política de promoção do AM é composta de amplo conjunto de ações que inclui práticas institucionais, como a Iniciativa Hospital Amigo da Criança (IHAC), a capacitação de profissionais do Sistema Único de Saúde (SUS), a criação e expansão da rede de bancos de leite humano e a Estratégia Amamenta e Alimenta Brasil, na promoção do AM e da alimentação complementar saudável pela atenção primária em saúde^2^. Destacam-se campanhas educativas intensivas na mídia e o engajamento da sociedade civil, com organizações como a Rede Internacional em Defesa do Direito de Amamentar, que fiscalizam a implementação das políticas e apoiam a promoção da amamentação. A adoção de normas e legislações específicas, como a Norma Brasileira de Comercialização de Alimentos para Lactentes e Crianças de Primeira Infância, Bicos, Chupetas e Mamadeiras e a regulamentação da licença-maternidade, complementa esse conjunto de ações^7,8^.

A literatura mostra que políticas e práticas de apoio no local de trabalho materno (espaços de lactação e intervalos para extração de leite), implementação da IHAC, contato pele a pele, método mãe canguru, alimentação com copo em ambientes de saúde e continuidade dos cuidados e apoio em ambientes comunitários e familiares são ações que contribuem para proteger, promover e apoiar a amamentação em larga escala^7,9,10^.

Também é conhecida a influência negativa da alimentação pré-láctea (APL), que é a introdução de quaisquer líquidos ou alimentos que não leite materno durante os primeiros três dias de vida sobre o AME até os seis meses^11,12^, entretanto o impacto de práticas hospitalares sobre a continuidade da amamentação foi pouco explorado, com resultados contraditórios^12–14^.

Diante desse cenário, buscamos investigar a associação entre práticas hospitalares na assistência ao nascimento e a manutenção do AM e do AME aos 2 meses de idade, além de compreender os principais motivos para a introdução da APL. A hipótese central é que determinadas condutas assistenciais adotadas nos hospitais, como a oferta de APL sem indicação clínica, podem impactar negativamente a continuidade do AM e do AME.

## MÉTODOS

Trata-se de uma coorte de base hospitalar e abrangência nacional, com o primeiro seguimento aos 2 meses de idade (Nascer no Brasil II: Pesquisa Nacional sobre Aborto, Parto e Nascimento), no período 2021–2024.

### Pesquisa Nascer no Brasil II

A população da Pesquisa Nascer no Brasil II (NBII) corresponde ao conjunto de mulheres internadas por motivo de parto (de nascido vivo ou natimorto) ou aborto, em hospitais com 100 ou mais nascidos vivos (NV) ao ano, segundo o Sistema de Informações sobre Nascidos Vivos (Sinasc). Foram excluídas as pacientes com parto trigemelar, as com dificuldade de comunicação (que não entendiam português, com deficiência auditiva, com doença mental severa) e as internadas para interrupção de parto por determinação judicial.

Foi selecionada uma amostra probabilística em dois estágios:

- Hospitais;
- Mulheres e seus recém-nascidos (RN).

Os hospitais foram estratificados por macrorregião, localização (região metropolitana ou interior), tipo (público, misto, privado) e tamanho (100–499, ≥ 500 NV/ano).

Nos hospitais selecionados, as mulheres admitidas para assistência ao parto foram identificadas pelos registros disponíveis no hospital: censo hospitalar e livro de internações. Após a identificação inicial, elas foram registradas numa lista única, conforme a ordem cronológica da data do parto, e selecionadas consecutivamente, até se atingir o número da amostra — 30 puérperas nos estabelecimentos com 100–499 NV/ano e 50 naqueles com ≥ 500 NV/ano. As mulheres foram abordadas durante a internação em seu próprio leito hospitalar.

A entrevista hospitalar incluiu informações sobre identificação, escolaridade, renda, antecedentes obstétricos, dados antropométricos, assistência pré-natal, morbidades, uso de medicamentos, intenção de amamentar, trabalho de parto e avaliação da assistência recebida. Além disso, foram fotografados e extraídos dados da caderneta da gestante e de exames de ultrassonografia obstétrica.

Dos prontuários hospitalares da mulher e do RN, foram extraídos dados sobre a assistência pré-natal; admissão hospitalar; assistência ao trabalho de parto, ao parto e ao nascimento; medicamentos e intervenções realizadas; morbidade materna e neonatal; internação em unidade de terapia intensiva (UTI); e condições de alta da mulher e do concepto. A coleta deu-se após a alta hospitalar ou até o 42º dia de internação da mulher e depois da alta hospitalar ou até o 28º dia de internação do RN.

Todas as puérperas entrevistadas na maternidade que autorizaram o contato posterior foram convidadas a participar do seguimento de dois meses. Essa entrevista abordou questões sobre: morbidade materna após a alta da maternidade; utilização de serviço de saúde ambulatorial; saúde do bebê; amamentação; satisfação com o atendimento; transtorno de estresse pós-traumático; vínculo mãe–bebê; depressão pós-parto; e ansiedade, conduzida por ligação telefônica ou por meio de *link* de autopreenchimento enviado pelo WhatsApp, com a utilização da plataforma Research Electronic Data Capture da Fundação Oswaldo Cruz.

Para a avaliação da estrutura e dos processos assistenciais dos serviços de obstetrícia e neonatologia, foram entrevistados os gestores das maternidades e os coordenadores da obstetrícia, da neonatologia, da epidemiologia e da farmácia. Os protocolos da etapa hospitalar e seguimentos da NBII se encontram publicados em Leal et al.^15^ e Miranda Theme-Filha et al.^16^.

### Estudo do Estado do Rio de Janeiro

A amostra de puérperas do estado do Rio de Janeiro foi calculada com base na proporção de cesarianas no estado em 2019 (57%), nível de significância de 5% e potência de 90% para detectar diferenças de 7%. Aplicamos um efeito de desenho de 1,3, resultando no planejamento de um tamanho amostral mínimo de 1.350 puérperas. Para alcançar esse quantitativo, ampliamos a amostra de puérperas (de 50 para 90) nos hospitais públicos e mistos com ≥ 500 NV/ano do estado do Rio de Janeiro. Cálculos *post-hoc* indicam que essa amostra tem poder de detectar diferenças absolutas de 5% para desfechos com prevalências entre 5 e 25%, equivalente a *odds ratio* (OR) superiores a 1,3. No estado do Rio de Janeiro, as entrevistas hospitalares ocorreram entre novembro/2021 e junho/2023 em 29 hospitais de 18 municípios.

### Critérios de Exclusão

Para a presente análise, foram excluídas as mulheres internadas por motivo de aborto e as com pelo menos um critério de risco (impeditivo ou dificultador) para a amamentação: óbito fetal ou neonatal, parto gemelar, idade gestacional < 34 semanas, peso ao nascer < 2.000 g, escore de Apgar aos 5 minutos < 8, RN internados em unidade de internação/UTI, e mulheres com o vírus da imunodeficiência humana (HIV) positivo. Além disso, excluímos os bebês com > 75 dias de vida no seguimento. Os RN que permaneceram na análise são referidos como RN saudáveis.

### Ponderação e Calibração Amostral

Foram aplicados pesos amostrais básicos, calibração da pesquisa de base (entrevista hospitalar) e calibração para as perdas de seguimento (não resposta). Os pesos amostrais básicos foram calculados pelo produto dos inversos das probabilidades de inclusão em cada estágio da amostra. A calibração da pesquisa de base ajustou a distribuição dos NV segundo características hospitalares (localização, tipo e tamanho) e das mulheres (idade e tipo de parto), com base nos dados do Sinasc de 2022. Para a calibração para as perdas de seguimento, modelamos a probabilidade de resposta como uma função das variáveis obtidas na pesquisa de base e usamo-las para derivar ajustes de peso de não resposta para as mulheres que responderam ao seguimento.

### Variáveis Desfecho

Analisamos o AM e o AME (não, sim) aos 2 meses de idade por meio de quatro perguntas de recordatório de 24 horas:

- De ontem de manhã até hoje de manhã, seu bebê mamou no peito?;
- De…, seu bebê tomou outro leite?;
- De…, seu bebê tomou água, chá ou suco?;
- De…, seu bebê comeu algum outro alimento?

### Variáveis de Exposição Principais

O contato pele a pele na sala de parto, o AM na primeira hora de vida e o AM nas primeiras 24 horas de vida foram relatados pelas puérperas durante a entrevista realizada no hospital. Já a APL na alta hospitalar e o apoio para o AM pós-alta foram relatadas pelas puérperas no primeiro seguimento. Consideramos como APL na alta hospitalar quando os RN não saíram da maternidade em AME. Todas as variáveis foram dicotomizadas (não, sim).

### Demais Variáveis de Exposição/Covariáveis

As características hospitalares incluíram a classificação dos hospitais em relação à IHAC em três categorias:

- Hospitais com a IHAC plenamente implementada;
- Hospitais em processo de habilitação;
- Hospitais que não se enquadravam em nenhuma das categorias.

Além disso, foi considerada a presença de alojamento conjunto exclusivo (não, sim), caracterizada quando o hospital dispunha exclusivamente de quartos pré-parto, parto e pós-parto, ou leitos obstétricos com alojamento conjunto. Ambas as informações foram obtidas por meio de entrevistas com os gestores dos estabelecimentos.

Como características sociodemográficas, incluímos a fonte de pagamento do parto (pública, privada); cor da pele (branca, preta, parda, asiática e indígena); idade (< 20, 20–34, ≥ 35 anos); anos de estudo (≤ 8, 9–11, 12–15, ≥ 16 ou ≥ 12); vive com companheiro (não, sim); trabalho remunerado (não, sim); e paridade (nulípara, um ou dois partos, ≥ três partos) ou (0–dois partos, ≥ três partos). Diferentes categorias para anos de estudo e paridade foram necessárias para se adequar a cada desfecho.

Consideramos como tendo fonte de pagamento pública as mulheres com parto em hospitais públicos ou privados conveniados ao SUS sem pagamento da internação por plano de saúde, e como tendo fonte de pagamento privada as com parto em hospitais privados e pagamento por plano de saúde ou desembolso direto.

Como dados do pré-natal e parto, incluímos se recebeu informação no pré-natal sobre a importância de amamentar na primeira hora de vida e sobre a prática de AME até os 6 meses (não: 0 ou apenas uma informação, sim: as duas informações); intenção de amamentar (< 6 meses, 6 meses a < 1 ano, 1 a < 2 anos, ≥ 2 anos); idade gestacional no nascimento em semanas completas (34–36, 37–38, ≥ 39) e tipo de parto (vaginal, que inclui fórceps e vácuo extrator; cesariana). As características sociodemográficas e do pré-natal foram relatadas pelas puérperas durante a entrevista hospitalar. A idade gestacional foi calculada preferencialmente com base em ultrassonografia precoce (realizada entre 6 e 13 semanas gestacionais), conforme registrado nos prontuários hospitalares e nas cadernetas das gestantes. O tipo de parto e as variáveis utilizadas para o critério de risco foram extraídos dos prontuários hospitalares.

### Análise dos Dados

As análises descritivas dos dados foram estratificadas pela fonte de financiamento do parto e do tipo de parto. Estimamos as proporções e os valores p do teste χ^2^ de Pearson. Para a análise dos fatores associados ao AM e AME, utilizamos regressão logística. Aplicamos um modelo teórico para classificar as variáveis de acordo com o seu nível hierárquico:

- Distais (nível 1): características hospitalares, sociodemográficas e paridade;
- Intermediárias 1 (nível 2): características do pré-natal e do parto;
- Intermediárias 2 (nível 3): práticas hospitalares;
- Proximal (nível 4): apoio pós-alta hospitalar para o AM.

Em cada nível, as variáveis foram selecionadas para inclusão no modelo ajustado com base no critério de p < 0,20, com exceção da fonte de financiamento do parto, tipo de parto e idade gestacional ao nascimento, que, por causa da extrema relevância^17^, foram incluídas no modelo independentemente do valor p. Além delas, foram mantidas no modelo final de regressão as variáveis que apresentaram associação significativa (p < 0,05) com os desfechos, após ajuste pelas variáveis do mesmo nível e de níveis hierárquicos superiores. Foram calculadas as razões de chance bruta e ajustada, juntamente com seus intervalos de confiança de 95% (IC95%). Por fim, a análise dos motivos para a APL foi estratificada pela fonte de financiamento do parto, tipo de parto e idade gestacional no nascimento, sendo estimadas as proporções e seus IC95%. Todas as análises foram realizadas com o *software* IBM Statistical Package for the Social Sciences Statistics for Windows, Versão 23.0 (IBM Corp., Armonk, NY, Estados Unidos da América), com inclusão do efeito de desenho, ponderação e calibração da amostra.

## RESULTADOS

Foram entrevistadas 1.762 puérperas nas maternidades do estado do Rio de Janeiro, das quais 225 (13%) foram excluídas com base no critério de risco. Entre as 1.537 puérperas restantes, houve 372 perdas de seguimento e 167 recusas, resultando na taxa de seguimento de 65%. Por fim, foram excluídos 39 bebês com mais de 75 dias de vida no seguimento. Dessa forma, a presente análise incluiu 959 mães e bebês com idades entre 45 e 75 dias, que foram calibrados para representar as 1.537 puérperas da linha de base. A idade média dos bebês foi de 61 dias, com percentis 25 e 75 de 56 e 65 dias, respectivamente, sem diferenças pelas variáveis de exposição e desfechos (dados não mostrados).

Observamos diferenças significativas nas características hospitalares e sociodemográficas segundo o financiamento do parto e o tipo de parto. Enquanto 51% das mulheres do setor público tiveram parto em hospitais com a IHAC implementada, no setor privado foram apenas 17%. O alojamento conjunto exclusivo foi altamente prevalente, abrangendo mais de 85% dos casos em ambos os tipos de financiamento. As mulheres com partos financiados pelo setor privado e as que tiveram cesariana tendem a ser mais brancas, mais velhas, com maior nível de educação, a terem trabalho, a viver com companheiro(a) e a terem menos filhos (Tabela 1).

**Tabela 1:** Características hospitalares e sociodemográficas das mulheres segundo financiamento do parto e tipo de parto. Pesquisa Nascer no Brasil II no estado do Rio de Janeiro, 2021–2023.

			Financiamento do parto				Tipo de parto		Total
			Público	Privado	Valor p^a^	Vaginal	Cesariana	Valor p^a^	
Total de mulheres			1.131	406		634	903		1.537
Idade no seguimento (dias)									
	45 a 60		48,2	49,1	0,848	50,4	47,2	0,399	48,6
	61 a 75		51,8	50,9		49,6	52,8		51,4
Características hospitalares									
	Iniciativa Hospital Amigo da Criança								
		Não	10,6	15,9		9,4	13,9		12,0
		Em processo^b^	38,4	67,1	0,04	35,0	53,6	0,03	46,0
		Sim	51,0	17,0		55,6	32,5		42,0
	Alojamento conjunto exclusivo								
		Não	9,7	12,5	0,77	5,3	14,1	0,32	10,5
		Sim	90,3	87,5		94,7	85,9		89,5
Características Sociodemográficas									
	Cor da pele								
		Branca	23,4	51,6		27,6	33,1		30,8
		Preta	25,5	9,4	< 0,001	23,5	19,6	< 0,001	21,2
		Parda	51,2	39,0		48,9	47,3		48,0
	Idade da mãe (anos)								
		10 a 19	13,6	1,7		15,4	6,9		10,4
		20 a 34	73,7	68,0	< 0,001	72,3	72,1	< 0,001	72,2
		35 ou mais	12,7	30,4		12,2	21,0		17,4
	Escolaridade (anos)								
		≤ 8 (ensino fundamental incompleto)	18,8	1,0		18,7	10,9		14,1
		9 a 11 anos (ensino fundamental completo)	35,7	8,4	< 0,001	31,9	26,1	< 0,001	28,5
		12 a 15 anos (ensino médio completo)	40,3	43,1		42,1	40,3		41,1
		≥ 16 anos (ensino superior completo)	5,1	47,5		7,3	22,6		16,3
	Vive com companheiro(a)								
		Não	22,9	5,7	< 0,001	22,7	15,3	< 0,001	18,3
		Sim	77,1	94,3		77,3	84,7		81,7
	Trabalho								
		Não	62,6	32,6	< 0,001	63,2	48,7	< 0,001	54,7
		Sim	37,4	67,4		36,8	51,3		45,3
	Paridade								
		Primípara	39,5	57,1		40,9	46,4		44,2
		Um ou dois partos anteriores	48,5	39,4	< 0,001	44,3	47,4	< 0,001	46,1
		Três ou mais partos anteriores	12,0	3,5		14,8	6,2		9,7

a Teste χ^2^.

b Hospitais em processo de habilitação para a Iniciativa Hospital Amigo da Criança ou com protocolos atualizados para atendimento a recém-nascidos na sala de parto, incluindo contato pele a pele precoce e aleitamento materno.

Orientações pré-natais sobre AM foram recebidas por pouco mais da metade das mulheres (52,9%), sendo mais frequentes no setor privado (64,1%). Mais de 40% pretendiam amamentar além de 2 anos e 60% por pelo menos um ano. O contato pele a pele na sala de parto e a amamentação na primeira hora de vida foram por volta de 60%, entretanto o AM nas primeiras 24 horas de vida e o AME na alta hospitalar aumentaram para quase 90%. As práticas hospitalares foram mais prevalentes no setor público e em partos vaginais. Quase 85% das mulheres se sentiram apoiadas na amamentação, e 95,2% amamentavam aos 2 meses de idade, sendo 61,4% exclusivamente, sem diferenças por tipo de parto ou financiamento (Tabela 2).

**Tabela 2:** Orientação, intenção, práticas hospitalares, apoio pós-alta e aleitamento materno aos 2 meses de idade, segundo financiamento do parto e tipo de parto. Pesquisa Nascer no Brasil II no estado do Rio de Janeiro, 2021–2023.

			Financiamento do parto			Tipo de parto			Total
			Público	Privado	Valor p^a^	Vaginal	Cesariana	Valor p^a^	
Total de mulheres			1.131	406		634	903		1.537
Recebeu orientação sobre amamentação no pré-natal									
	Não		51,1	35,9	0,047	51,1	44,3	0,124	47,1
	Sim		48,9	64,1		48,9	55,7		52,9
Intenção de amamentar									
	Sim		96,6	98,0	0,190	96,2	97,4	0,162	96,7
		< 6 meses	4,7	3,3		3,7	4,8		4,3
		6 meses a < 1 ano	26,1	27,3		28,6	24,8		26,4
		1 a < 2 anos	19,0	16,6	0,581	18,4	18,3	0,524	18,3
		≥ 2 anos	41,9	42,2		39,9	43,4		42,0
	Não pensou sobre o tempo		8,3	10,6		9,4	8,7		9,0
Práticas hospitalares									
	Contato pele a pele na sala de parto		57,8	66,1	0,232	80,7	45,4	< 0,001	60,0
	Aleitamento materno na primeira hora de vida		65,6	51,7	0,041	78,3	50,2	< 0,001	61,9
	Aleitamento materno nas primeiras 24 horas de vida		88,7	88,2	0,887	92,9	85,4	< 0,001	88,5
	Alimentação pré-lactea na alta hospitalar		11,0	20,4	0,028	10,3	15,6	0,044	13,4
Apoio para o amamentar pós-alta hospitalar									
	Não		16,3	12,9	0,374	14,8	15,9	0,623	15,4
	Sim		83,7	87,1		85,2	84,1		84,6
Aleitamento materno aos 2 meses^b^									
	Sim		94,8	96,5		96,0	94,7		95,2
		Exclusivo	60,9	64,2		64,0	60,2		61,4
		Não exclusivo	33,9	32,3	0,303	32,0	34,5	0,683	33,8
	Não		5,2	3,5		4,0	5,2		4,8

a Teste χ^2^.

b Aleitamento materno entre 45 e 75 dias de vida, média e mediana de 61 dias de vida.

A chance de AM foi maior entre mulheres que deram à luz em hospitais IHAC (OR = 2,35; IC95% 1,04–5,32), naquelas com ≥ 12 anos de estudo (OR = 1,96; IC95% 1,08–3,58), com três ou mais partos anteriores (OR = 4,35; IC95% 1,06–17,83) e que tinham a intenção de amamentar ≥ um ano (OR = 1,58; IC95% 1,09–2,31). A APL na alta hospitalar se associou negativamente ao AM (OR = 0,04; IC95% 0,02–0,13). Já as mulheres que se sentiram apoiadas após a alta tiveram maior chance de AM (OR = 6,91; IC95% 3,57–13,35) (Tabela 3).

**Tabela 3:** Fatores associados ao aleitamento materno aos 2 meses de idade. Pesquisa Nascer no Brasil II no estado do Rio de Janeiro, 2021–2023.

			Aleitamento materno^a^		Valor p^b^	Regressão ajustada^c^	
			Não (%)	Sim (%)		OR	IC95%
Total de mulheres (n = 1.537)			4,8	95,2	-	-	-
	Idade no seguimento (dias)						
		45 a 60	4,9	95,1	0,922	ref	-
		61 a 75	5,0	95,0		0,98	0,62–1,54
Nível 1							
	Financiamento do parto						
		Público	5,0	95,0	0,647	1,64	0,56–4,76
		Privado	4,4	95,6		ref	-
	Iniciativa Hospital Amigo da Criança						
		Não	7,9	92,1		ref	-
		Em processo^d^	4,6	95,4	0,196	2,04	0,86–4,81
		Sim	4,2	95,8		2,35	1,04–5,32
	Alojamento conjunto exclusivo						
		Não	6,8	93,2	0,119	ref	-
		Sim	4,6	95,4		1,73	0,94–3,30
	Cor da pele						
		Branca	5,0	95,0		ref	-
		Preta	5,9	94,1	0,662	0,87	0,18–4,11
		Parda	4,1	95,9		1,32	0,71–2,44
	Idade da mãe (anos)						
		10 a 19	4,5	95,5		1,61	0,13–0,63
		20 a 34	5,4	94,6	0,483	ref	-
		35 ou mais	2,9	97,1		1,78	0,56–5,70
	Escolaridade (anos)						
		≤ 8	6,6	93,4		ref	-
		9 a 11	4,6	95,4	0,550	1,66	0,36–7,64
		≥ 12 anos	4,4	95,6		1,96	1,08– 3,58
	Vive com companheiro(a)						
		Não	5,2	94,8	0,870	ref	-
		Sim	4,8	95,2		1,08	0,31–3,77
	Trabalho						
		Não	5,3	94,7	0,465	ref	-
		Sim	4,3	95,7		1,39	0,72–2,69
	Paridade						
		0 a dois partos anteriores	5,2	94,8		ref	-
		Três ou mais partos anteriores	1,7	98,3	0,200	4,35	1,06–7,83
Nível 2							
	Recebeu orientações sobre amamentação no pré-natal						
		Não	5,3	94,7	0,680	ref	-
		Sim	4,5	95,5		1,09	0,53–2,26
	Intenção de amamentar por um ano ou mais						
		Não	6,3	93,7	0,004	ref	-
		Sim	3,9	96,1		1,58	1,09–2,31
	Tipo de parto						
		Vaginal	4,1	95,9	0,463	1,37	0,55–3,43
		Cesariana	5,4	94,6		ref	-
	Idade gestacional (semanas)						
		34–36	1,4	98,6		4,06	0,45–37,07
		37–38	3,9	96,1	0,157	1,46	0,83–2,56
		≥ 39	5,7	94,3		ref	-
Práticas hospitalares (nível 3)							
	Contato pele a pele logo após o nascimento						
		Não	6,1	93,9	0,313	ref	-
		Sim	3,9	96,1		1,60	0,61–4,19
	Aleitamento materno na primeira hora de vida						
		Não	5,5	94,5	0,595	ref	-
		Sim	4,4	95,6		0,75	0,25–2,19
	Aleitamento materno nas primeiras 24 horas de vida						
		Não	7,1	92,9	0,377	ref	-
		Sim	4,5	95,5		1,02	0,30–3,48
	Alimentação pré-lactea na alta hospitalar						
		Não	2,3	97,7	< 0,001	ref	-
		Sim	21,1	78,9		0,04	0,02– 0,13
Nível 4							
	Apoio para a amamentação pós-alta hospitalar						
		Não	14,5	85,5	< 0,001	ref	-
		Sim	2,8	97,2		6,91	3,57–13,35

IC95%: intervalo de confiança de 95%.

OR: *odds ratio*.

APL: alimentação pré-láctea.

ref: referência.

a Aleitamento materno entre 45 e 75 dias de vida, média e mediana de 61 dias de vida.

b Teste χ^2^.

c Nível 1 ajustado pelo financiamento do parto, Iniciativa Hospital Amigo da Criança, escolaridade e paridade. Nível 2 e Nível 3 ajustados pelo financiamento do parto, Iniciativa Hospital Amigo da Criança, escolaridade, paridade, intenção de amamentar um ano ou mais, tipo de parto e idade gestacional. Nível 4 ajustado pelo financiamento do parto, Iniciativa Hospital Amigo da Criança, escolaridade, paridade, intenção de amamentar um ano ou mais, tipo de parto, idade gestacional e APL na alta hospitalar.

d Hospitais em processo de habilitação para a Iniciativa Hospital Amigo da Criança ou com protocolos atualizados para atendimento a recém-nascidos na sala de parto, incluindo contato pele a pele precoce e aleitamento materno.

A chance de AME foi maior nas mulheres com financiamento público do parto (OR = 1,33; IC95% 1,02–1,72), nas com ≥ 16 anos de estudo (OR = 2,27; IC95% 1,10–4,71), que viviam com companheiro(a) (OR = 1,33; IC95% 1,06–1,67) e que tinham intenção de amamentar ≥ um ano (OR = 1,49; IC95% 1,05–2,11). A APL na alta hospitalar se mostrou negativamente associada ao AME (OR = 0,15; IC95% 0,09–0,28) (Tabela 4).

**Tabela 4:** Fatores associados ao aleitamento materno exclusivo aos 2 meses de idade. Pesquisa Nascer no Brasil II no estado do Rio de Janeiro, 2021–2023.

			Aleitamento materno exclusivo^a^		Valor p^b^	Regressão ajustada^c^	
			Não (%)	Sim (%)		OR	IC95%
Total de mulheres (n = 1.537)			38,6	61,4	-	-	-
	Idade no seguimento (dias)						
		45 a 60	41,5	58,5	0,248	ref	-
		61 a 75	35,7	64,3		1,27	0,83–1,96
Nível 1							
	Financiamento do parto						
		Público	38,9	61,1	0,653	1,33	1,02–1,72
		Privado	37,5	62,5		ref	-
	Iniciativa Hospital Amigo da Criança						
		Não	36,3	63,7		ref	-
		Em processo^d^	38,7	61,3	0,819	0,87	0,56–1,33
		Sim	39,1	60,9		0,91	0,57–1,44
	Alojamento conjunto exclusivo						
		Não	40,0	60,0	0,799	ref	-
		Sim	38,4	61,6		0,97	0,57–1,65
	Cor da pele						
		Branca	41,2	58,8		ref	-
		Preta	38,3	61,7	0,540	1,15	0,81–1,63
		Parda	37,0	63,0		1,22	0,82–1,82
	Idade da mãe (anos)						
		10 a 19	48,0	52,0		0,76	0,47–1,24
		20 a 34	37,9	62,1	0,242	ref	-
		35 ou mais	35,8	64,2		1,10	0,65–1,85
	Escolaridade (anos)						
		≤ 8	46,9	53,1		ref	-
		9 a 11	43,0	57,0	0,047	1,11	0,61–2,03
		12 a 15	35,3	64,7		1,61	0,84–3,09
		≥ 16	31,5	68,5		2,27	1,10–4,71
	Vive com companheiro(a)						
		Não	46,5	53,5	0,001	ref	-
		Sim	36,9	63,1		1,33	1,06–1,67
	Trabalho						
		Não	39,1	60,9	0,764	ref	-
		Sim	37,9	62,1		0,91	0,68–1,21
	Paridade						
		Primípara	39,4	60,6		1,20	0,74–1,94
		Um ou dois partos anteriores	35,2	64,8	0,074	1,57	0,86–2,85
		Três ou mais partos anteriores	50,8	49,2		ref	-
Nível 2							
	Recebeu orientações sobre amamentação no pré-natal						
		Não	39,3	60,7	0,768	ref	-
		Sim	37,9	62,1		1,04	0,79–1,37
	Intenção de amamentar por um ano ou mais						
		Não	45,0	55,0	0,028	ref	-
		Sim	34,5	65,5		1,49	1,05–2,11
	Tipo de parto						
		Vaginal	35,4	64,6	0,401	1,43	0,83–2,47
		Cesariana	40,7	59,3		ref	-
	Idade gestacional (semanas)						
		34–36	38,2	61,8		0,95	0,53–1,69
		37–38	39,3	60,7	0,942	0,93	0,70–1,24
		≥ 39	38,2	61,8		ref	-
Práticas hospitalares (nível 3)							
	Contato pele a pele logo após o nascimento						
		Não	42,5	57,5	0,335	ref	-
		Sim	35,6	64,4		1,23	0,70–2,17
	Aleitamento materno na primeira hora de vida						
		Não	41,2	58,8	0,275	ref	-
		Sim	36,7	63,3		1,07	0,78–1,47
	Aleitamento materno nas primeiras 24 horas de vida						
		Não	46,1	53,9	0,151	ref	-
		Sim	37,5	62,5		1,23	0,75–2,01
	Alimentação pré-lactea na alta hospitalar						
		Não	32,9	67,1	< 0,001	ref	-
		Sim	75,1	24,9		0,15	0,09–0,28
Nível 4							
	Apoio para a amamentação pós-alta hospitalar						
		Não	42,2	57,8	0,450	ref	-
		Sim	37,7	62,3		1,20	0,78–1,86

IC95%: intervalo de confiança de 95%; OR: *odds ratio*; APL: alimentação pré-láctea; ref: referência.

a Aleitamento materno exclusivo entre 45 e 75 dias de vida, média e mediana de 61 dias de vida.

b Teste χ^2^.

c Nível 1 ajustado pelo financiamento do parto, escolaridade, vive com companheiro e paridade. Nível 2 e Nível 3 ajustados pelo financiamento do parto, escolaridade, vive com companheiro, paridade, intenção de amamentar por um ano ou mais, tipo de parto e idade gestacional. Nível 4 ajustado pelo financiamento do parto, escolaridade, vive com companheiro, paridade, intenção de amamentar por um ano ou mais, tipo de parto, idade gestacional e APL na alta hospitalar.

d Hospitais em processo de habilitação para a Iniciativa Hospital Amigo da Criança ou com protocolos atualizados para atendimento a recém-nascidos na sala de parto, incluindo contato pele a pele precoce e aleitamento materno.

A APL na alta hospitalar foi mais frequente no setor privado (20,4%), nos nascidos entre 37 e 38 semanas completas (18,3%) denominados termo precoce, e nas cesarianas (15,6%). O principal motivo relatado pelas puérperas foi leite insuficiente e/ou dificuldades na pega, representando mais de 70% dos casos (Tabela 5).

**Tabela 5:** Prevalência e motivos da alimentação pré-láctea na alta hospitalar segundo financiamento do parto, tipo de parto e idade gestacional no nascimento, Pesquisa Nascer no Brasil II no estado do Rio de Janeiro, 2021–2023.

		Financiamento		Tipo de parto		Idade gestacional em semanas completas			Total
		Público	Privado	Vaginal	Cesariana	34–36	37–38	≥ 39	
		1.131	406	634	903	98	503	936	1.537
Total de mulheres		%	%	%	%	%	%	%	%
		(IC95%)	(IC95%)	(IC95%)	(IC95%)	(IC95%)	(IC95%)	(IC95%)	(IC95%)
Alimentação pré-láctea na alta hospitalar		11,0 (8,6–13,9)	20,4 (14,4–28,1)	10,3 (7,3–14,3)	15,6 (12,5–19,2)	10,5 (3,7–26,3)	18,3 (14,3–23,0)	11,1 (9,0–13,6)	13,4 (11,1–16,1)
Motivos									
	RN apresentou problema de saúde	0,8 (0,5–1,6)	2,1 (1,0–4,5)	1,1 (0,4–2,7)	1,2 (0,6–2,4)	1,9 (0,5–7,6)	1,3 (0,6–2,7)	1,0 (0,5–2,1)	1,1 (0,7–1,9)
	Puérpera apresentou problema de saúde	0,6 (0,3–1,5)	1,7 (0,5–5,3)	0,3 (0,1–1,2)	1,3 (0,6–2,9)	0,0 -	0,7 (0,2–1,8)	1,1 (0,4–2,7)	0,9 (0,4–1,9)
	Leite insuficiente e/ou dificuldades na pega	7,8 (5,2–11,6)	14,5 (10,2–20,2)	7,3 (4,7–11,1)	11,1 (8,4–14,3)	7,0 (2,8–16,3)	12,6 (9,1–17,3)	8,1 (6,0–10,9)	9,5 (7,4–12,1)
	Rotina hospitalar	1,0 (0,3–2,9)	1,7 (0,6–4,6)	1,0 (0,3–3,3)	1,2 (0,5–2,8)	0,0 -	2,4 (0,9–6,2)	0,6 (0,2–2,1)	1,1 (0,5–2,5)
	Outro motivo	2,6 (1,7–3,9)	4,5 (2,4–8,2)	2,9 (1,7–4,9)	3,2 (1,9–5,2)	2,2 (0,6–7,5)	5,9 (3,8–9,3)	1,6 (0,8–3,5)	3,0 (2,2–4,2)

IC95%: intervalo de confiança de 95.

RN: recém-nascido.

## DISCUSSÃO

Características hospitalares, sociodemográficas, de intenção e apoio ao AM se mostraram de extrema relevância para a continuidade do AM. O contato pele a pele na sala de parto e a amamentação na primeira hora de vida ocorreram com menor frequência do que esperado. Em contrapartida, a amamentação nas primeiras 24 horas foi quase universal, com redução na diferença entre os tipos de parto. Nenhuma das três práticas mostrou associação com a continuidade do AM. Por sua vez, a APL na alta hospitalar reduziu a chance do AM e do AME aos 2 meses de idade.

Em países de baixa e média renda^11^, bem como em crianças amazônicas de Cruzeiro do Sul (Acre)^12^, a APL encurtou a duração do AME e aumentou a probabilidade de consumo de fórmulas em crianças com menos de 6 meses. Além do impacto negativo da APL, constatamos que produção de leite insuficiente e/ou dificuldades na pega do bebê foi o principal motivo relatado pelas mães de RN saudáveis do estado do Rio de Janeiro. Em todo o mundo cerca de metade das mães relata a percepção de leite insuficiente como a principal razão para a introdução de alimentos para bebês nos primeiros meses de vida e para a interrupção prematura do AM, o que geralmente pode ser evitado ou tratado com sucesso com o suporte adequado^9^.

Não só a APL, mas também o motivo de a APL ser produção de leite insuficiente e/ou dificuldades na pega do bebê foi maior no setor privado, nos nascimentos termo precoce e nas cesarianas. A cesariana já se mostrou associada à APL em países da América Latina e Caribe^18^ e a taxas mais baixas de diversos indicadores de AM, como início precoce^19,20^, AM na alta hospitalar^20^ e AME antes dos 6 meses de vida^20^. Estudos citam a dor no pós-operatório^21,22^, dificuldades na mobilidade^22^ e início tardio da produção de leite como principais causas do atraso na amamentação após cesarianas^21,23,24^. O Brasil apresenta taxas extremamente elevadas de cesariana (58% segundo o Sinasc de 2022) e de nascimentos a termo precoce (aproximadamente 35%), ambos associados à menor chance de AM na primeira hora de vida e de AME na internação hospitalar^17^. Nesse contexto, priorizar o início precoce do AM juntamente com o suporte físico e emocional de profissionais de saúde e de familiares é essencial para elevar os níveis de ocitocina e prolactina, fortalecendo a confiança das mães e o vínculo mãe–bebê.

A importância da IHAC no aumento das taxas de amamentação é evidente^20^. Mulheres que tiveram parto em hospitais IHAC no estado do Rio de Janeiro apresentaram maior chance de manter o AM, embora algumas práticas não tenham se mostrado isoladamente associadas. Esse achado sugere que outras dimensões da IHAC, como a oferta restrita de APL, serviços de apoio durante a internação e encaminhamento para grupos de apoio pós-alta, desempenham papel essencial na continuidade do AM. Um estudo prospectivo em Xangai salientou que mães que receberam serviços de apoio de qualidade durante a internação apresentaram maior probabilidade de manter o AME tanto na alta hospitalar quanto aos 6 meses^25^. De forma semelhante, uma pesquisa com puérperas norte-americanas mostrou que hospitais que seguem ao menos seis dos dez passos da IHAC aumentam significativamente as chances de AME, destacando o papel institucional dessas práticas^26^.

Mães com partos financiados pelo setor público apresentaram maior chance de manter o AME. No setor público, por causa da maior frequência da IHAC, há menor influência das indústrias de fórmulas infantis, o que reduz a prescrição de substitutos do leite materno. Além disso, o acesso ao suporte contínuo de equipes multiprofissionais e programas comunitários, como a Estratégia Saúde da Família e a Estratégia Amamenta e Alimenta Brasil, podem ter contribuído para esse achado.

A escolaridade materna e a intenção de amamentar por pelo menos um ano se revelaram como variáveis de extrema importância, o que pode ser explicado pelo melhor acesso a informações sobre práticas de amamentação e seus benefícios, maior acesso aos serviços de saúde, maior autonomia na tomada de decisões e maior apoio familiar e social, destacando o papel das atitudes e intenções pré-natais no comportamento de amamentação. Um estudo sobre conhecimento, práticas e atitudes encontrou que o conhecimento sobre amamentação, atitude, norma subjetiva e controle de prática esteve positivamente relacionado ao AME aos quatro meses, sendo barreiras comuns a falta de confiança na amamentação; dificuldades com a lactação; e a percepção equivocada de suprimento inadequado de leite^27^. Uma revisão sistemática concluiu que comportamentos instáveis do bebê, especialmente o choro persistente, podem levar os pais a acreditarem que a suplementação com fórmulas infantis seja necessária^28^.

Mulheres com alta paridade apresentaram maior probabilidade de manter o AM, sugerindo que a experiência prévia na maternidade e amamentação facilita o sucesso do AM atual, possivelmente por aumento do conhecimento, maior confiança e estratégias mais eficazes para superar desafios^29^. Já o AME foi menos prevalente nessas mulheres, embora sem significância estatística. Uma hipótese seria a maior dificuldade de manejo do AME com um número maior de filhos, que exigiria mais tempo e organização.

A convivência com um(a) companheiro(a) se mostrou associada à maior probabilidade de AME, mas não ao AM. Esse achado sugere que o suporte no núcleo familiar pode desempenhar um papel crucial na manutenção do AME, prevenindo a introdução de outros líquidos ou alimentos além do leite materno. O AME, ao exigir uma adaptação rigorosa da rotina familiar, pode demandar maior comprometimento e alinhamento entre os membros da família. Em contrapartida, mulheres que se sentiram apoiadas apresentaram maior prevalência de AM, o que não se refletiu no AME. Uma possível explicação é que o apoio percebido esteja relacionado a redes externas ao núcleo familiar, como apoio social ou de profissionais e serviços de saúde, eficazes em prevenir o desmame precoce, mas com menor impacto na manutenção do AME, que exige uma adaptação diária mais intensa.

Entre os pontos fortes do estudo, destacamos o desenho e o tamanho amostral, que asseguram a representatividade do estado do Rio de Janeiro; e a padronização da coleta de dados e a baixa taxa de recusa no hospital. Todos contribuíram para a robustez dos resultados. Como limitação, apontamos para o momento de coleta das variáveis de exposição APL na alta hospitalar e apoio para o AM pós-alta hospitalar, simultâneo à aferição dos desfechos, o que pode ter introduzido um viés de causalidade reversa.

Concluímos que a política de promoção do AM de amplo espectro, implementada no Brasil, tem sido eficaz na quase universalização do AM aos 2 meses de idade em RN saudáveis no estado do Rio de Janeiro. No entanto, para aumentar a prevalência do AME, é necessário avançar em várias áreas. As prioridades identificadas neste estudo incluem: fornecer informações baseadas em evidências e livres de influência da indústria às mulheres e suas famílias no pré-natal, parto e puerpério, com atenção especial a grupos vulneráveis (baixa escolaridade, primíparas e sem companheiro); implementar programas para reduzir cesarianas especialmente antes das 39 semanas; capacitar gestores e profissionais de saúde para apoiar e manejar dificuldades na amamentação durante a internação para o parto e no pós-parto; e ampliar a adesão às práticas hospitalares de promoção da amamentação, incluindo a regulamentação do uso de APL, especialmente no setor privado.

Para pesquisas futuras, recomenda-se aprofundar a investigação sobre os fatores que influenciam a APL, considerando não apenas a prática hospitalar, mas também a percepção materna de insuficiência de leite e dificuldades na pega. Pesquisas também devem explorar estratégias eficazes para reduzir as barreiras à amamentação em contextos específicos, como cesarianas e partos a termo precoce e prematuros, e avaliar o papel de redes de apoio (familiares e comunitárias) na continuidade do AME. Por fim, é essencial investigar a efetividade de programas públicos de suporte à amamentação, considerando sua implementação no setor privado, e o impacto de políticas de regulação sobre a prescrição e distribuição de fórmulas infantis.

## Data Availability

Os conjuntos de dados gerados e/ou analisados durante o estudo estão disponíveis com o autor correspondente mediante solicitação.
